# National Use of Asbestos in Relation to Economic Development

**DOI:** 10.1289/ehp.0901196

**Published:** 2009-09-29

**Authors:** Giang Vinh Le, Ken Takahashi, Antti Karjalainen, Vanya Delgermaa, Tsutomu Hoshuyama, Yoshitaka Miyamura, Sugio Furuya, Toshiaki Higashi, Guowei Pan, Gregory Wagner

**Affiliations:** 1 Department of Environmental Epidemiology, University of Occupational and Environmental Health, Kitakyushu, Japan;; 2 Finnish Institute for Occupational Health, Helsinki, Finland;; 3 Japan Occupational Safety and Health Center, Tokyo, Japan;; 4 Department of Work, Systems and Health, University of Occupational and Environmental Health, Kitakyushu, Japan;; 5 Liaoning Provincial Centre for Disease Prevention and Control, Shenyang, People’s Republic of China;; 6 National Institute for Occupational Safety and Health, Washington, DC, USA

**Keywords:** asbestos diseases, asbestos use, economic development, environmental Kuznets curve, income level, inflection points

## Abstract

**Background:**

National disparities in asbestos use will likely lead to an unequal burden of asbestos diseases.

**Objectives:**

As economic status may be linked to asbestos use, we assessed, globally, the relationship between indicators of national economic development and asbestos use.

**Methods:**

For the 135 countries that have ever used asbestos, per capita asbestos use (kilograms per capita per year) was compared with per capita gross domestic product (GDP) in 1990 Geary–Khamis dollars (GKD) for the period 1920–2003. Countries were grouped into three income levels (high, middle, and low) that were adapted from the 2003 World Bank categories.

**Results:**

The historical pattern of asbestos use followed the environmental Kuznets curve in which use by high-income countries peaked when incomes attained 10,000–15,000 GKD and essentially ceased at income levels over 20,000 GKD. Currently, middle- and low-income countries are increasing their use of asbestos, closely following the paths once traced by higher income countries.

**Conclusions:**

Developing countries have the opportunity to eliminate asbestos use sooner than high-income countries and thus reduce the future burden of asbestos diseases.

An estimated 90,000 asbestos-related deaths occur worldwide every year, and 125 million people are occupationally exposed to asbestos (Driscoll et al. 2005a, 2005b; Concha-Barrientos et al. 2004). The World Health Organization (WHO 2006) and the International Labour Organization (ILO 2006) have recommended that the best way to eliminate asbestos disease is to cease asbestos use. Many countries have substantially reduced or banned asbestos use because of increasing public health problems. Indeed, the bulk of countries with the greatest historic consumption of asbestos have largely retreated from its current use.

Transitions to abandon asbestos use have been achieved mostly in high-income countries, whereas use is still common (Lancet 2008), to variable degrees, in many developing countries (Takahashi and Karjalainen 2003). Efforts to transition may be hampered in developing countries because of ignorance, misinformation and aggressive marketing by exporters (Joshi and Gupta 2004), as well as a false sense of reassurance caused by long latency period before disease manifestation, and inadequate surveillance systems.

Recently, the Conference of Parties for the Rotterdam Convention failed to reach an agreement on including chrysotile, the predominant type of asbestos in use today, in the list of Prior Informed Consent (PIC) [United Nations (UN), United Nations Environmental Programme (UNEP), Food and Agriculture Organization of the United Nations (FAO) 2008]. As other types of asbestos are already on the PIC list, the preclusion of chrysotile contradicts the recommendations by the WHO and the ILO, which explicitly called to stop using all types of asbestos. In effect, exporting countries are exempted from the need to obtain consent from importing countries, the majority of which are in early developmental stages. Chrysotile exemption from the PIC list may expand asbestos use by poorer countries.

Given the pending threat of a global epidemic of asbestos disease (Peto et al. 1995, 1999), disparities among countries in the level and pattern of asbestos use warrant in-depth analyses, as they plausibly forecast an unequal burden of disease.

In this study, we provide a global analysis of the use of asbestos by countries over time and relate it to a standard measure of economic development.

## Materials and Methods

The U.S. Geological Survey (USGS) maintains an open report on worldwide supply and consumption trends of asbestos in raw or fiber form (Virta 2006). In the appendix of that report, the USGS presents compiled data, by country, on production, import, export, and consumption of asbestos in 10-year intervals from 1920 to 1960, in 5-year intervals from 1970 to 1995, and annually from 1996 to 2003. The report has been widely accessed for quantitative assertions of the asbestos situation at global and national levels. Consumption (or use) is defined as production plus import minus export.

Per capita asbestos use (measured in kilograms per capita per year) is a useful indicator to compare the state of asbestos use among countries (LaDou 2004; Tossavainen et al. 1997). Recently, we and others demonstrated the potential of this indicator to serve as a surrogate measure for the general exposure level of a population, which can also be used to estimate subsequent health burdens at national levels (Antao et al. 2009; Lin et al. 2007; Nishikawa et al. 2008; Takahashi et al. 1999). In this paper, we adopt the USGS definition of use but consider negative use values (resulting from storage, for example) to be uninformative and exclude such data from further analysis.

Economic development is assessed by per capita gross domestic product (GDP), which is measured in units of International Geary–Khamis 1990 dollars (GKD). GKD are estimated by converting currencies into a common unit, based on the twin concepts of purchasing power parity of currencies and international average prices (Maddison 1995; UN 1992) to enable comparisons across countries and over time. The GKD database of Angus Maddison (Maddison 2001, 2008) covers most countries and has been widely applied in long-term economic growth analysis. GKD has also been recently applied in an empirical exploration of the relationship between national income and sulfur dioxide emissions (Markandya et al. 2006).

We analyzed the national circumstances of 135 countries with available data on both asbestos use and per capita GDP. Countries were grouped into 3 income levels according to the World Bank (2003) categories: high (*n* = 28), middle (*n* = 63; upper-middle and lower-middle levels were merged), and low income (*n* = 40). Countries and entities with asbestos data but lacking information on income category by the World Bank, such as the former Union of Soviet Socialist Republics (USSR), Czechoslovakia, Yugoslavia, and Taiwan, were grouped as unclassified. Individual countries in the different income groups were selected for graphical presentation based on population size in 2003 (≥ 10 million) to ensure adequate representation of continents and income groups and to feature countries with high asbestos use and continuity of data.

We weighted means by the size of national populations whenever we calculated group means. We obtained population data from the WHO (2009), the U.S. Census Bureau (2008), and Laymeyer (2006), prioritized in that order. Data were compiled using Microsoft Excel (Microsoft Corporation, Redmond, WA, USA). Graphs were drawn using SigmaPlot (version 9.01.; Systat Software Inc., San Jose, CA, USA). Names of countries were abbreviated according to the International Organization for Standardization alpha-3 code (UN 2009).

**Figure 1:**
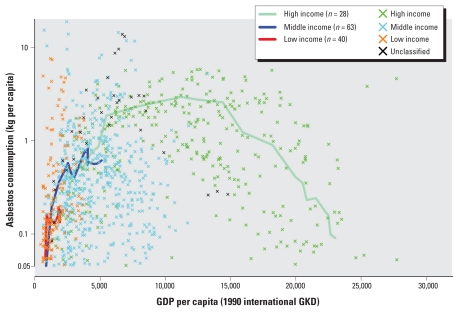
Asbestos use versus GDP in the world.

**Figure 2:**
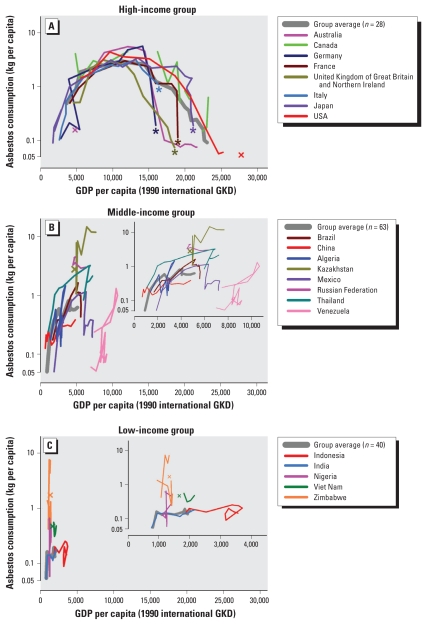
Asbestos use versus GDP by income level groups. Symbols: ×, data point that cannot be connected with line graph because of presence of adjacent data point with value < 0.05 kg per capita; *, no data or data < 0.05 kg per capita because of ban. Looping patterns occur when asbestos use and income status reversed trends during the time course.

Data points with per capita use < 0.05 kg/capita/year were included in calculations of group means but were omitted from the graphical presentation (Figure 1). Similarly, individual country trends, shown as line graphs in Figure 2, were necessarily interrupted when per capita use was < 0.05 kg/capita/year or when data were lacking. The cutoff value of 0.05 kg/capita/year corresponds to 500 (5,000) tons/year in countries with populations of 10 (100) million persons. It is necessary to bear in mind the USGS reservation that consumption patterns in countries using < 5,000 tons/year were too erratic to ascertain any trends in asbestos use.

## Results

Worldwide, the 135 countries that have ever used asbestos and for which economic data are available cumulatively used 181 million tons during the period of 1920–2003, apportioned as 48%, 22%, 4%, and 26% to high-, middle-, low-income, and unclassified groups, respectively (Table 1). These countries, on average, used 0.79 kg/capita/year during this period and by income group, 1.58, 0.40, 0.11, and 4.14 kg/capita/year, respectively. In the unclassified group, the former USSR alone recorded a cumulative use of 42.8 million tons (24% of world) and an annual per capita use of 4.95 kg/capita/year. Table 1 also shows that lower-income countries have fewer tendencies to ratify the ILO Asbestos Convention (ILO 1986), report pleural cancer and/or mesothelioma to the WHO, and ban the use of asbestos.

Figure 1 shows all historical data points recorded for the 135 countries in terms of per capita asbestos use vis-à-vis per capita GDP for the same year. Connected lines represent the trend (or rates in terms of Δuse per ΔGDP where Δ represents change) in weighted averages of the two variables for the three income groups. Collectively, countries moved along a curved trajectory, with the lower-income group generally trailing the path of the higher-income group. Whereas high-income countries showed clear peaks that then approached nil after sustained downtrends, middle- and low-income countries showed upward trends, with a steeper rate for the latter. The middle- and low-income groups surpassed the high-income group in 1990–1995 and 1999, respectively [see Supplemental Material, Figure 1, available online (doi:10.1289/ehp.0901196.S1 via http://dx.doi.org/)], reaching 0.62, 0.16, and 0.09 kg/capita/year in 2003, respectively (Table 1). These values correspond to the right end points of each trajectory in Figure 1.

High-income countries (Figure 2A) generally showed parallel use patterns: steady increases from approximately 5,000 GKD, to peaks at approximately 10,000 GKD, sustained to approximately 15,000 GKD, followed by variable downtrends. Note that peaks at approximately 10,000–15,000 GKD were formed in different years ranging from 1950 to 1995 [see Supplemental Material, Figure 2 (doi:10.1289/ehp.0901196.S1)]. Collectively, asbestos use tended to flatten to near zero over 20,000 GKD. Among middle-income countries (Figure 2B), Venezuela made a clear downturn at ca. 10,000 GKD. Other middle-income countries exhibit mixed trends at lower GKD levels: gradual upward (e.g., China), downward (e.g., Mexico and Brazil), or indiscernible (e.g., Kazakhstan, Russia, and Thailand). Similarly, low-income countries (Figure 2C) show mixed trends: fluctuating (e.g., Zimbabwe), sustaining higher than group average (e.g., Viet Nam), or steady (e.g., India and Indonesia). Note that looping patterns occurred when asbestos use and income status reversed trends during the time course (e.g., Venezuela and Indonesia).

## Discussion

The global historical pattern of national asbestos use vis-à-vis per capita GDP is consistent with the so-called environmental Kuznets curve (EKC). The trajectories of individual high- income countries are remarkably similar despite different time courses. A common ceiling or inflection point in asbestos use is observed at an income level of approximately 10,000–15,000 GKD, in line with the EKC theory.

The EKC theory postulates an inverted U-shaped relationship between environmental pollutant levels and economic growth (Andreoni and Levinson 2001; Grossman and Krueger 1995; World Bank 1992): examples include SO_2_, NO_x_, and lead (in air) and sewage (in water). Unlike the situation with by-products of or emissions from industrial processes, which may be compounded by the lack of comparable definitions and/or data, reliable historical statistics are available for asbestos, a longstanding industrial commodity. By applying a per capita indicator, we assessed asbestos use trends over 8 decades in most countries of the world.

The figures show no time dimension, but countries in fact move along a common time axis, experiencing simultaneous economic development and, up to a point, asbestos use. To show the bivariate relationship over time, Motion Chart, a web-based software application designed for tracking several data points to see changes over time (Google 2009), was applied to the data set [see Supplemental Material, Figure 3 (doi:10.1289/ehp.0901196.S1). A clear log-linear to log-curvilinear relationship between per-capita GDP and asbestos use is observed during earlier years. This relationship begins to collapse when countries consecutively peak out use at the inflection point.

The wax-and-wane use pattern exhibited by high-income countries is probably associated with acceptance, over time, of the fact that asbestos is an established carcinogen. The final step to abandon asbestos use appears to have become easier with the increasing availability of safer and commercially viable substitutes. In contrast, middle- and low-income countries continue or even increase use with economic growth at the respective stages of development. It is plausible to assume that countries with a long history of high asbestos use (and thus a high accumulation of asbestos in the society) have already seen the disease burden taking its toll, whereas those with a short history have not or have only started to see diseases reflecting recent use.

The positive correlation between asbestos use and GDP observed prior to the inflection point suggests interdependence between the two factors. During times of soaring infrastructure demands, intense forces for use of inexpensive construction materials are in play. The subsequent downturn in use occurs despite continued economic growth. Moreover, high-income countries did not sustain use and eventually shifted to abandonment. Even Canada, a major producer and exporter of asbestos, appears to follow the path common to high-income countries, although major fluctuations are evident. Societal responses to hazards of asbestos (ultimately bans for all types of asbestos including chrysotile) have been embraced by high-income countries but notably less so by lower-income countries.

Inflection points in consumption at approximately 10,000–15,000 GKD were experienced by most high-income countries/entities with a few exceptions (e.g., United Arab Emirates and Hong Kong) [see Supplemental Material, Figure 2 (doi:10.1289/ehp.0901196.S1). This point was also observed for Venezuela, a middle-income country approaching a higher-income level. Since the dissolve of the USSR in 1991, Russia and Kazakhstan have recorded asbestos use at 3.53 and 7.82 kg/capita/year, respectively, which contributed to the overall high group mean of the middle-income countries. The collective path of the middle-income group may be tapering slightly, indicating early signs of deviation from the trailing path. This could arise because of a perceived “benefit [arising] from the science and engineering lessons of the early movers” (Levinson 2008). However, the ultimate responses to current knowledge and the experience of high-income countries is uncertain.

As our group means were weighted by national population sizes, group patterns will most strongly reflect trends in populous countries (e.g., China and India). These countries have moderate per-capita use levels because of their large populations, and they exhibit steady trends. However, even a moderate level of per capita use in such countries indicates a high total use (in 2003, China and India consumed 492,000, and 192,000 tons, respectively), which can potentially lead to the exposure of many people. The application of income per capita assessed in GKD reflected only one aspect of economic development, but nevertheless allowed a comparison of countries on a global historical scale.

## Conclusions

In summary, empirical data on asbestos use suggest that *a*) high-income countries followed paths in which asbestos use correlated with increasing economic development until an inflection point of around 10,000–15,000 GKD; *b*) high-income countries, beyond 20,000 GKD, have completed transitions to nonuse; and *c*) middle- and low-income countries are currently increasing asbestos use, closely following paths once traced by higher-income countries. High asbestos use by high-income countries was not sustained. Each developing country is at a crossroad, with an opportunity to choose an earlier reduction and elimination in use and thus a reduction in future disease burden. The scientific community has a vital role to play in assisting such transitions and in the transfer of technologies for the prevention of asbestos diseases.

## Figures and Tables

**Table 1:** Asbestos use in the world and groups of countries/entities, by recent income level.

		Group by income level^a^ (*n*, countries)			
	World (135)	Low (40)	Middle (63)	High (28)	Unclassified^b^ (4)
Use (1920–2003)					
Cumulative use as group [10^6^ ton] (% of world)	180.82 (100.0)	7.46 (4.1)	39.22 (21.7)	87.58 (48.4)	46.53 (25.7)
Annual use per capita, country mean^c^ [kg/capita/year]	0.79	0.11	0.40	1.58	4.14
Historical peak [kg/capita/year] (year)	1.27 (1980)	0.19 (2000)	0.82 (1997)	2.95 (1970)	10.71 (1985)
In year 2003 [kg/capita/year]	0.36	0.16	0.62	0.09	NA
No. of countries ratified ILO Convention^d^ (% of column total)	28 (20.7)	2 (5.0)	12 (19.0)	14 (50.0)	NA
No. of countries reported mortality^e^ to WHO (% of column total)	62 (45.9)	5 (12.5)	32 (50.8)	25 (89.3)	NA
No. of countries banned use (% of column total)	40 (29.6)	0 (0)	19 (30.2)	21 (75.0)	NA

NA, not applicable.

a Classified by World Bank in 2003 as low (≤ 735 $US), lower middle (736–2,935 $US), upper middle (2,936–9,075 $US), and high (≥ 9,076 $US) according to 2002 gross national income (GNI) per capita. Note that the time-trend analyses in figures are based on the application of the GKD as an indicator of GDP per capita.

b Four countries/entities without World Bank data on category of income level are former USSR (dissolved 1991), Czechoslovakia (split 1993), Yugoslavia (disintegrated 1992), and Taiwan. The former USSR alone used 42.84 million tons, 23.7% of the world’s total, at 4.95 kg/capita/year during the observed period.

c Country mean is weighted by size of national population.

d ILO Convention on Asbestos (ILO 2009).

e ICD-9 163 (malignancy of the pleura) and ICD-10 C45 (mesothelioma). Status as of February 2009 for ratification, reporting mortality, and banning use.
